# Processing speed impairment after anterior communicating artery aneurysm rupture assessed using the wechsler adult intelligence scale: an observational study

**DOI:** 10.1186/s12883-026-04902-9

**Published:** 2026-04-15

**Authors:** Takuya Okui, Natsuko Otani, Takamitsu Iwata, Koichi Beppu, Ryo Horii, Ryuichiro Kajikawa, Takashi Tsuzuki

**Affiliations:** 1 Department of Neurosurgery, Sakai City Medical Center, 1-1 Ebaraji-cho, 1-chome, Nishi-ku, Sakai, Osaka 593-8304 Japan; 2 Department of Rehabilitation Technology, Sakai City Medical Center, 1-1 Ebaraji-cho, 1-chome, Nishi-ku, Sakai, Osaka 593-8304 Japan

**Keywords:** Subarachnoid hemorrhage, Anterior communicating artery aneurysm, Processing speed, Neurocognitive impairment, Wechsler Adult Intelligence Scale

## Abstract

**Background:**

Subarachnoid hemorrhage (SAH) commonly causes subtle, but functionally disabling, cognitive impairment. Although prior studies have emphasized the mechanisms of diffuse brain injury, the relationship between aneurysm location and domain-specific cognitive deficits remains unclear. Processing speed is a pivotal factor influencing post-SAH functional outcomes; however, it remains poorly understood, although it is known to depend on the integrity of the anterior cingulate cortex (ACC), located adjacent to the anterior communicating artery (Acom). This study investigated whether co-ruptured SAH selectively impairs processing speed using the Wechsler Adult Intelligence Scale (WAIS).

**Methods:**

Twenty-nine patients with SAH (August 2016 to August 2025) underwent neuropsychological evaluation using the WAIS-III/IV, including five indices: the Full-Scale IQ (FSIQ), Verbal Comprehension Index (VCI), Perceptual Reasoning Index (PRI), Working Memory Index (WMI), and Processing Speed Index (PSI). The Hasegawa Dementia Scale–Revised (HDS-R) was used as the screening measure. Multivariate linear regression was applied to examine predictors of PSI. Group comparisons between Acom and non-Acom SAH were conducted using Welch’s t-tests. Within-group differences were evaluated using the Friedman and Wilcoxon post-hoc tests. Interhemispheric hematoma thickness in the frontal horn-callosal rostrum (FH–CR) and Callosal Genu (CG) planes were quantified on admission CT and correlated with the PSI.

**Results:**

PSI was markedly lower (82.9 ± 12.6; mean ± SD) than other WAIS indices (FSIQ, VCI, PRI, and WMI). Regression analysis identified Acom aneurysm location as the only independent predictor of lower PSI (β = −15.68, *p* = 0.044). The Acom group demonstrated significantly reduced PSI compared with the non-Acom group (72.9 ± 9.95 vs. 88.11 ± 10.56, *p* = 0.00109; Welch’s t-test), with no differences in other indices. Hematoma thickness at both FH–CR and CG planes was significantly greater in the Acom group and correlated negatively with PSI (ρ = −0.626 and − 0.593, respectively).

**Conclusion:**

Acom-ruptured SAH is associated with a selective impairment in processing speed, likely reflecting the region-specific vulnerability of the anterior cingulate structures. WAIS assessment enables the sensitive detection of these deficits and may guide early targeted rehabilitation.

**Supplementary Information:**

The online version contains supplementary material available at 10.1186/s12883-026-04902-9.

## Background

Subarachnoid hemorrhage (SAH) is a devastating cerebrovascular disorder that accounts for approximately 5–10% of all strokes, with a global incidence of 8.3/100,000 person-years [1]. The anterior communicating artery (Acom) is the most common site of ruptured aneurysms, accounting for nearly 38% of cases [2]. Between 1990 and 2019, the worldwide prevalence of SAH among adults aged 20–54 years increased from 439.48 to 490.03 per 100,000 people [3], underscoring its substantial socioeconomic burden. Although advances in treatment reduced mortality by 3.98% between 1990 and 2021 [4], cognitive sequelae during the chronic phase remain a major concern. These impairments are often subtle, and may elude routine neurological or neuroimaging examinations [5, 6]; however they significantly hinder the return to work, financial and household management, and instrumental activities of daily living, thereby markedly diminishing patients’ quality of life [7, 8].

While numerous studies have described post-SAH cognitive impairment, most have focused on chronic-phase screening tools (e.g., Montreal Cognitive Assessment (MoCA), Frontal Assessment Battery, Trail Making Test (TMT)), and comprehensive neuropsychological evaluation during the acute phase remains limited. Given the demonstrated benefits of early cognitive rehabilitation after stroke [9, 10], timely identification of and intervention for neurocognitive and neuropsychiatric disturbances following SAH remain essential.

Cognitive deficits in SAH are generally attributed not to focal injury, but to diffuse brain dysfunction resulting from elevated intracranial pressure, hypoperfusion, impaired oxygenation, blood–brain barrier disruption, cerebral edema, and inflammatory responses [11–13]. Consequently, most reports indicate that there is no direct association between aneurysm location and domain-specific cognitive deficits [11, 14–16]. Although prior studies have assessed memory, executive dysfunction, and language, impairment of processing speed, an essential determinant of real-world functional recovery, has received far less attention [17, 18]. Although patient’s TMT performance reflects aspects of processing speed, this value is substantially confounded by visuospatial search, set-shifting, and working memory demands, limiting its specificity and robustness as a pure measure of processing speed [19, 20]. In contrast, the Processing Speed Index (PSI) of the Wechsler Adult Intelligence Scale (WAIS) is a highly standardized and reliable metric widely regarded as a valid indicator of processing efficiency [21, 22]. Processing speed is associated with activity in the anterior cingulate cortex (ACC) [23, 24], a brain region anatomically adjacent to the Acom complex, and is linked to susceptibility to blood-related mass effects in Acom-ruptured SAH.

Therefore, we hypothesized that Acom-ruptured SAH selectively impairs processing speed; this hypothesis was tested using measurement of the WAIS.

## Methods

Thirty-five patients diagnosed with SAH at our institution eligible for cognitive assessments between August 2016 and August 2025 were assessed. After excluding six patients with unidentified hemorrhage sources, 29 patients were included (see Table 1 for patient characteristics). Cognitive function was quantitatively assessed using the five principal indices of the WAIS-III or WAIS-IV, as follows: the Full-Scale Intelligence Quotient (FSIQ: overall intellectual ability), Verbal Comprehension Index (VCI: verbal conceptual reasoning and lexical knowledge), Perceptual Reasoning Index (PRI: visuospatial and nonverbal problem-solving abilities), Working Memory Index (WMI: attentional control and executive working memory), and Processing Speed Index (PSI: efficiency of visual information processing and graphomotor speed). The Hasegawa Dementia Scale–Revised (HDS-R), a widely used cognitive screening instrument, was administered [25]. All neuropsychological tests were administered by trained examiners (Speech-Language-Hearing Therapist) according to standardized procedures. Neuropsychological assessments were performed during hospitalization or early after transfer to a rehabilitation program, depending on each patient’s clinical stability and ability to complete testing. In our institutional workflow, the WAIS was administered after the high-risk period for symptomatic vasospasm had passed and only when sedation and delirium were absent and the patients were deemed able to perform the tasks reliably (i.e., stable general condition, adequate arousal and attention, and sufficient cooperation), rather than at a fixed post-onset day. Consequently, patients with lower initial severity (WFNS I–II) were typically assessed earlier, whereas some patients with higher severity underwent WAIS assessment after completion of a specialized stroke rehabilitation program. The interval from SAH onset to WAIS assessment was recorded for each patient and incorporated into the statistical analyses as a covariate.

**Table 1:** Patient characteristics

		*n* = 29
Sex	Male	14(48.3%)
	Female	15(51.7%)
Age	Mean	49.9 ± 11.2
	Range	25 ~ 78
WFNS grade	Grade Ⅰ	14(48.3%)
	Grade Ⅱ	6(20.1%)
	Grade Ⅲ	0(0.00%)
	Grade Ⅳ	5(17.2%)
	Grade Ⅴ	4(13.8%)
Aneurysm location	Anterior Communicating Artery	10(34.5%)
	Internal Carotid Artery	10(34.5%)
	Middle Cerebral Artery	3(10.3%)
	Vertebral Artery	3(10.3%)
	Anterior Cerebral Artery	1(3.4%)
	Posterior Cerebral Artery	1(3.4%)
	Basilar Artery	1(3.4%)
Treatment modality	Coil embolization	21(72.4%)
	Clipping	8(27.6%)
Time from onset to assessment	Mean	26.4 ± 18.6
	Range	10–78

The mean scores and pairwise correlations between the five WAIS indices and HDS-R were computed. For each WFNS grade, the difference between PSI and the mean of the other four indices was calculated, and the PSI was compared across the WFNS grades using one-way analysis of variance (ANOVA). A multivariate linear regression analysis was subsequently conducted with PSI as the dependent variable and age, WFNS grade, aneurysm location (Acom vs. non-Acom), time from onset to WAIS assessment, WAIS version (WAIS-III vs. WAIS-IV) as the independent variables. Group differences in the WAIS performance between the Acom and non-Acom cohorts were assessed using Welch’s t-test. Within the Acom group, the overall differences across the five WAIS indices were evaluated using the Friedman test, followed by post-hoc comparisons using the Wilcoxon signed-rank test to determine whether PSI was selectively reduced relative to the other four indices. The same analytical procedure was used for the non-Acom group.

Hematoma thickness along the interhemispheric fissure was measured on admission CT at two axial levels: the Frontal Horn–Callosal Rostrum (FH–CR) plane and the Callosal Genu (CG) plane. At each level, hematoma thickness was defined as the maximal perpendicular thickness of hyperdense clot along the interhemispheric fissure on that slice, measured in millimeters using the caliper tool. The same predefined anatomical landmarks were used across all cases. Measurements were independently performed by two blinded raters, and the mean of the two measurements was used for the subsequent analyses. Differences in hematoma thickness between the Acom and non-Acom groups were examined using the Mann–Whitney U test. The association between the PSI and hematoma thickness was investigated using Spearman’s rank correlation coefficients.

## Results

### WAIS and HDS-R scores and their correlations

**Fig. 1:**
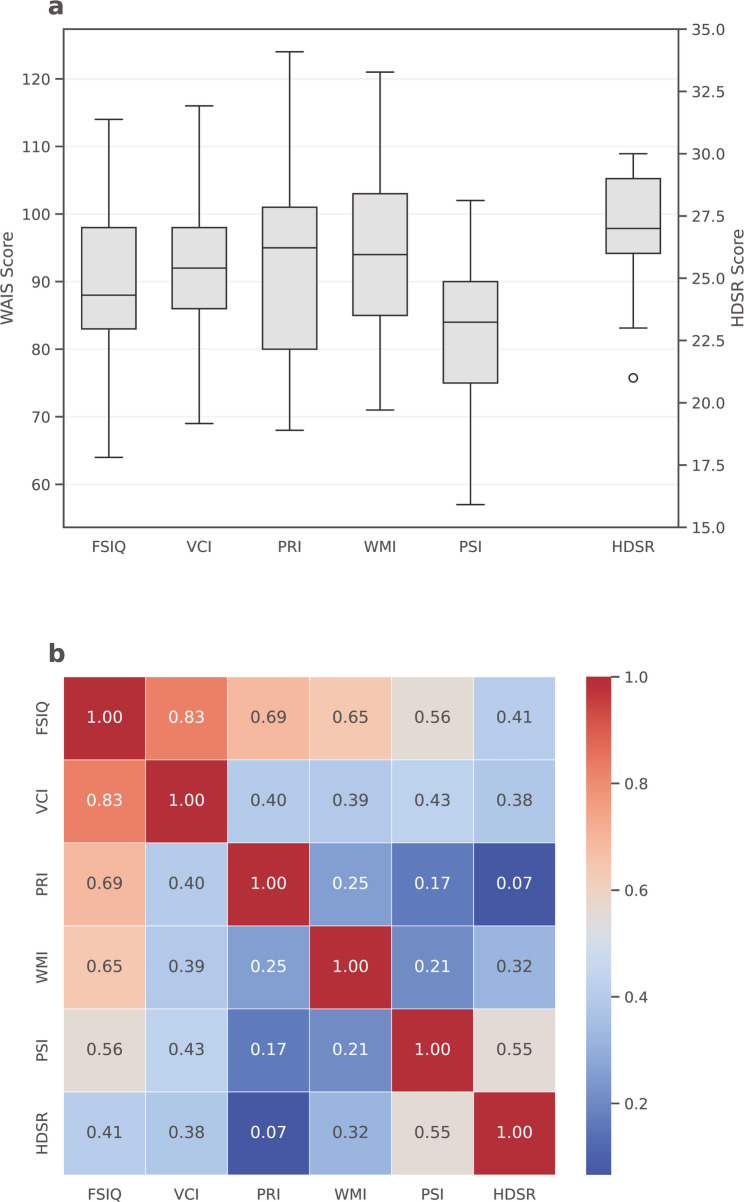
WAIS-IV Indices and HDS-R. **A** Box-and-whisker plots illustrating the score distributions of the five WAIS indices (FSIQ, VCI, PRI, WMI, PSI) alongside the Hasegawa Dementia Scale–Revised (HDS-R). The WAIS indices correspond to the left vertical axis, whereas the right vertical axis represents HDS-R scores. **B** Heatmap depicting the pairwise correlations among the WAIS indices (FSIQ, VCI, PRI, WMI, PSI) and HDS-R. Each cell displays the Pearson correlation coefficient, and the color gradient reflects the strength and direction of the association: warm colors indicate stronger positive correlations, while cool colors indicate weaker correlations

The mean scores for the five WAIS indices (FSIQ, VCI, PRI, WMI, and PSI) and HDS-R were calculated for the 29 enrolled patients. The mean FSIQ was 88.9 ± 11.6 (mean ± SD), indicating a mild reduction in overall intellectual functioning at the cohort level. VCI (92.9 ± 11.9), PRI (92.7 ± 13.4), and WMI (93.9 ± 12.2) all remained within the average range, whereas PSI was markedly lower (82.9 ± 12.6), demonstrating a characteristic decline in processing speed. The mean HDS-R score was 27.1 ± 2.36, with all patients exceeding the dementia cutoff (20> ) (Fig. 1A).

Pearson correlations showed a strong association between the FSIQ and VCI (*r* = 0.83, *p* < 0.001), and moderate correlations with the PRI (*r* = 0.69, *p* = 0.033), WMI (*r* = 0.65, *p* = 0.038), and PSI (*r* = 0.56, *p* = 0.022). The HDS-R further demonstrated a weaker, but significant, correlation with the FSIQ (*r* = 0.41, *p* = 0.027). The PRI showed the weakest cross-domain associations, particularly with the HDS-R (*r* = 0.07, *p* = 0.734) (Fig. 1B).

### Processing speed index across WFNS grades

**Fig. 2:**
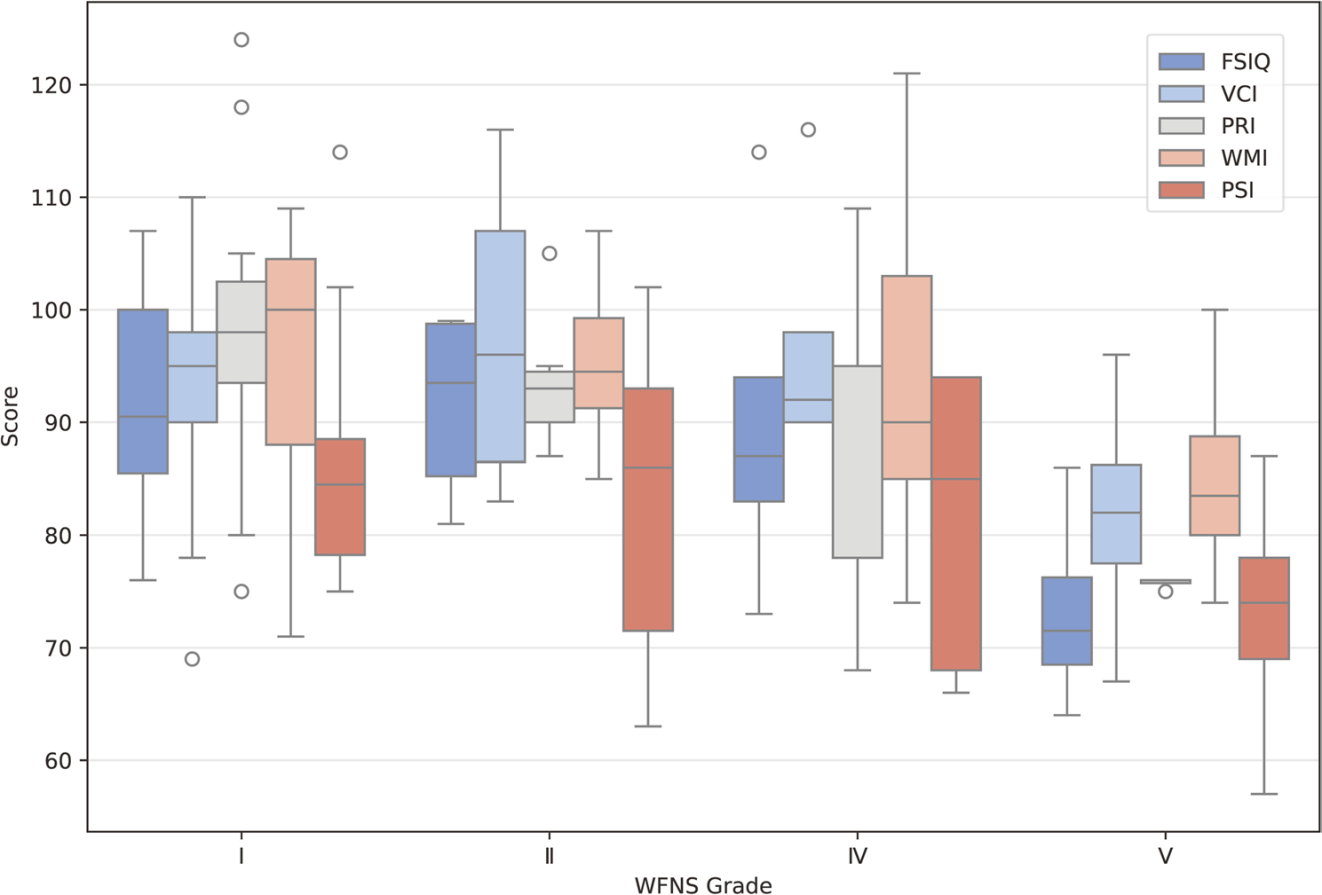
WAIS index scores by WFNS Grade. The distribution of WAIS index scores (FSIQ, VCI, PRI, WMI, PSI) stratified by WFNS grade (I, II, IV, and V). The X-axis denotes WFNS grade, and the Y-axis represents the standard scores for each WAIS index. WFNS grade III is not displayed because no patients classified as grade III were included in the study cohort

For each WFNS grade, the difference between PSI and the mean of the other four indices was computed. The difference values were − 8.87 ± 12.76 (WFNS I), − 11.38 ± 14.47 (WFNS II), − 11.35 ± 13.54 (WFNS IV), and − 6.00 ± 6.68 (WFNS V), indicating consistently lower PSI across all severity grades. A one-way ANOVA revealed no significant differences in PSI among the WFNS categories (F = 0.995, *p* = 0.411). (Fig. 2)

### Multivariable regression analysis for PSI

**Fig. 3:**
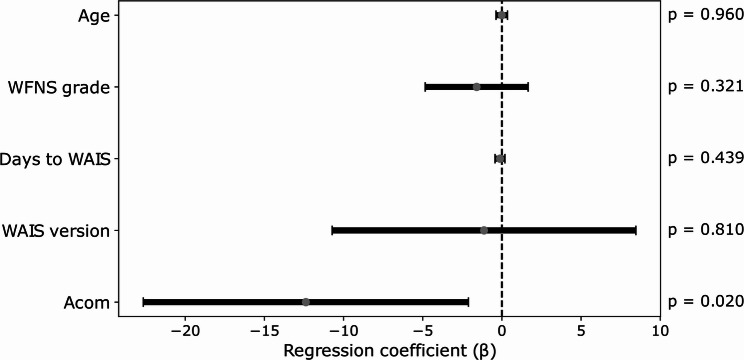
The coefficient values of the multivariable regression model

Multivariate linear regression analysis, with PSI as the dependent variable, demonstrated a significant overall model (R² =0.405, F = 3.692, *p* = 0.0134). Acom aneurysm location was the only significant negative predictor of PSI (β = −12.38, 95% CI − 22.648 to − 2.113, *p* = 0.0202). Age, WFNS grade, Days to WAIS, and WAIS version showed no significant associations. (Fig. 3; Table 2)

**Table 2:** Multivariable linear regression analysis for processing speed index

	β	SE	t	*p*	95%CI low	95%CI high	VIF
age	-0.009	0.176	-0.05	0.9604	-0.372	0.355	1.016
WFNS	-1.593	1.571	-1.014	0.3213	-4.843	1.658	1.573
Day to WAIS	-0.119	0.151	-0.788	0.4388	-0.433	0.194	2.085
WAIS version	-1.128	4.637	-0.243	0.81	-10.72	8.464	1.073
Acom	-12.38	4.963	-2.494	0.0202	-22.648	-2.113	1.517

### Comparison of wais indices between acom and non-acom groups

**Fig. 4:**
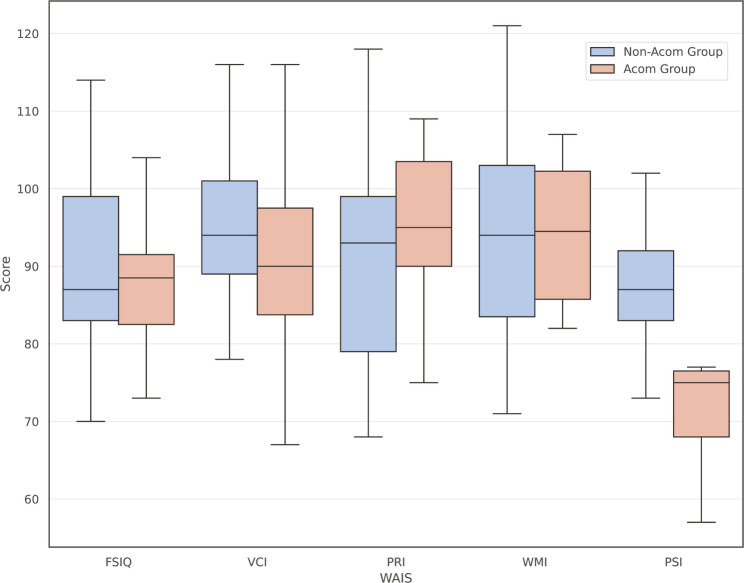
Comparison of WAIS between Acom group and non-Acom group. Box-and-whisker plots comparing WAIS index scores (FSIQ, VCI, PRI, WMI, PSI) between the Acom and non-Acom groups, classified according to the location of the ruptured aneurysm

Demographic and clinical characteristics of the Acom and non-Acom groups are shown in Table 3.　PSI was significantly lower in the Acom group compared with the non-Acom group (72.9 ± 9.95 vs. 88.11 ± 10.56; mean difference [Acom − non-Acom] = − 15.21, 95% CI − 23.51 to − 6.91; Hedges’ g = − 1.427; t = − 3.83, *p* = 0.00109; Welch’s t-test). No significant differences were observed in the remaining indices, including the FSIQ (*P* = 0.444), VCI (*P* = 0.506), PRI (*P* = 0.306), and WMI (*P* = 0.833) (Fig. 4, also see supplementary Table 1).

**Table 3:** Baseline characteristics of patients with acom versus non-acom aneurysms

	Acom	Non-Acom	P-value
Age	48.50 ± 12.07	50.63 ± 10.96	0.6469
WFNS grade	Ⅰ 4 Ⅱ 3 Ⅳ 1 Ⅴ 2	Ⅰ 10 Ⅱ 3 Ⅳ 4 Ⅴ 2	0.6232
Treatment modality			
Coil embolization	9 (90.0%)	14(73.7%)	0.303
Clipping	1 (10.0%)	5(26.3%)	
Time from onset to WAIS assessment	39.00 ± 25.31	19.74 ± 9.05	0.0415

### Post-hoc Comparison of PSI with Other WAIS Indices

In the Acom group, within-profile differences across the five WAIS indices (FSIQ, VCI, PRI, WMI, PSI) were first examined using the Friedman test, which demonstrated a significant overall effect (χ² = 19.07, *p* = 0.00076). Post-hoc Wilcoxon signed-rank tests revealed that PSI was significantly lower than FSIQ (W = 1.0, *p* = 0.0020), VCI (W = 0.0, *p* = 0.0010), PRI (W = 1.0, *p* = 0.0039), and WMI (W = 1.0, *p* = 0.0020), with all comparisons remaining significant after Bonferroni correction (α = 0.0125), indicating a selective decrement in processing speed relative to other cognitive domains.

In contrast, for the non-Acom group, the Friedman test did not show a significant overall difference across indices (χ² = 7.90, *p* = 0.095), and none of the post-hoc comparisons between PSI and the other indices met the Bonferroni-corrected threshold (PSI vs. FSIQ: W = 77.0, *p* = 0.2343; PSI vs. VCI: W = 51.0, *p* = 0.0382; PSI vs. PRI: W = 72.0, *p* = 0.1772; PSI vs. WMI: W = 50.5, *p* = 0.0636), providing no evidence for selective PSI impairment in this group.

### Impact of SAH-related hematoma burden on PSI

Next, we compared the interhemispheric hematoma thickness between the Acom and non-Acom groups using the mean values across the two raters’ measurements. Across all patients, the mean hematoma thickness was 3.38 ± 2.72 mm at the FH–CR plane and 4.53 ± 5.28 mm at the CG plane (see Supplementary Fig. 1). Thus, the mean hematoma thickness was significantly greater in the Acom group at both levels (FH–CR: 6.35 ± 1.92 mm vs. 1.82 ± 1.49 mm, *p* = 1.35 × 10⁻⁵; CG: 9.25 ± 5.95 mm vs. 2.05 ± 2.60 mm, *p* = 1.42 × 10⁻^4^, Mann–Whitney U tests).

**Fig. 5:**
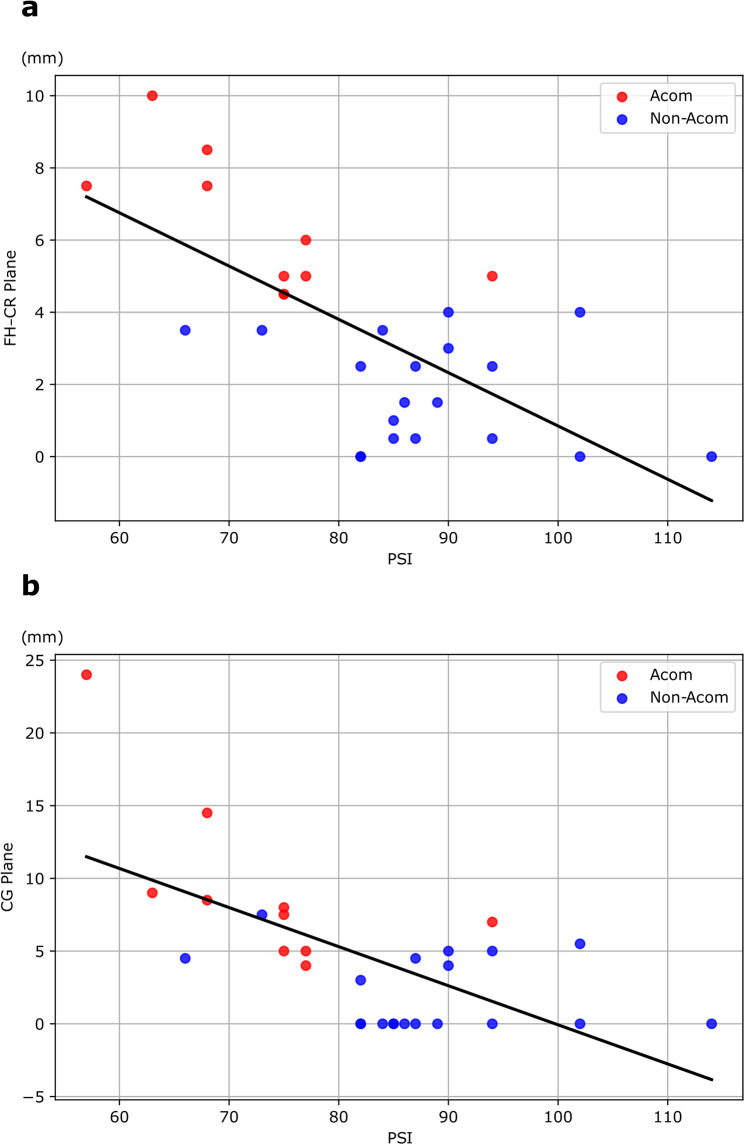
The relationship between PSI and hematoma thickness. Scatter plots illustrating the association between the Processing Speed Index (PSI) and hematoma thickness measured on the FH–CR Plane (**A**) or CG Plane (**B**). Red points represent Acom cases, blue points represent non-Acom cases, and the black lines indicate the linear regression line fitted to all subjects

To clarify the relationship between hematoma burden and processing speed, we subsequently examined the association between PSI and hematoma thickness, finding that PSI showed significant negative correlations with hematoma thickness at both the FH–CR plane (ρ = −0.602, *p* = 5.46 × 10⁻⁴, Spearman’s rank correlation) and the CG plane (ρ = −0.553, *p* = 1.87 × 10⁻³. These results indicate that a greater interhemispheric hematoma burden is closely linked to more pronounced impairment in processing speed. (Fig. 5)

## Discussion

Overall, this study demonstrated that processing speed is selectively impaired in patients with Acom aneurysm-ruptured SAH, as quantified by the WAIS PSI. The PSI reflects the capacity to rapidly and efficiently process visually presented information through simple or overlearned tasks, and impairment in this domain manifests as a slowed performance of activities such as document handling, transcription, and data verification, affecting daily, academic, and occupational functioning [19, 20]. Neurocognitive evidence has established that processing speed critically depends on ACC integrity [23, 24].

Traditionally, cognitive impairment following SAH has been attributed to diffuse brain injury secondary to elevated intracranial pressure, cerebral hypoperfusion, neuroinflammation, and blood–brain barrier dysfunction [11–13]. If the PSI decline was driven solely by these diffuse mechanisms, comparable impairment would be expected, regardless of the aneurysm location. However, only the Acom group demonstrated a significant PSI reduction, which was specific to processing speed, without any corresponding deficits in the FSIQ, VCI, PRI, or WMI domains. These findings strongly support a mechanism involving region-specific vulnerability, particularly in the ACC, which is anatomically adjacent to the Acom complex. Indeed, past functional MRI studies have shown that faster reaction times in simple response tasks are associated with greater ACC activation [26], and that the metabolic integrity of the ACC and prefrontal cortex correlates with processing speed and executive measures in Parkinson’s disease [27]. Furthermore, ACC and supplementary motor area activities contribute to response monitoring and error detection [28], and structural compromise in these regions, such as age-related white matter disease, has been linked to slow processing speeds [29]. Together, these data indicate that processing speed relies on the functional and structural integrity of a cognitive control network centered on the ACC and prefrontal cortex. Furthermore, the interhemispheric and callosal hematoma thicknesses were significantly greater in the Acom group, and correlated negatively with PSI, indicating that the mass effect and local inflammatory or ischemic injury involving the cingulate structures may contribute to slowed processing.

As the time from onset to WAIS assessment varied across patients, we conducted additional analyses to evaluate whether assessment timing influenced PSI. We observed a negative association between time-to-assessment and PSI (see Supplementary Fig. 2). Importantly, however, the timing difference appeared to reflect confounding by initial severity in our cohort: patients with more severe SAH were more likely to undergo WAIS assessment after completion of a specialized rehabilitation program, resulting in later testing (see Supplementary Fig. 3). Consistent with this interpretation, in multivariable regression including time-to-assessment as a covariate, assessment timing did not independently contribute to PSI, whereas Acom aneurysm location remained the only significant negative predictor (see Supplementary Fig. 4).

Importantly, a reduced processing speed was observed even in patients with low-grade WFNS I–II SAH, indicating that subtle cognitive sequelae may remain undetected on routine neurological examinations. Consequently, early cognitive assessment incorporating PSI is essential, particularly for Acom lesions, to identify deficits that may hinder the return to work or independent living. Although the TMT-B could serve as a pragmatic alternative when WAIS administration is impractical, it captures a broader mixture of processes, including set-shifting, visuospatial search, and motor execution, making it less specific to processing speed [19, 30]. Screening tools such as the HDS-R or Mini-Mental State Examination lack the sensitivity to detect such subtle deficits [31]. Consistently, all patients in the present cohort scored within the normal range on the HDS-R scale. The MoCA is a brief, high-sensitivity screening battery that samples multiple domains, including memory, attention, language, abstract reasoning, visuospatial ability, and executive function and is considered superior for identifying mild cognitive impairment [32]. However, unlike the WAIS, it does not allow fine-grained, domain-specific quantification, and is therefore suboptimal for a detailed analysis of processing speed. Overall, the WAIS remains the most sensitive and domain-specific tool for detecting processing speed impairments after SAH, and should be incorporated when feasible.

This study was limited by its single-center, retrospective design, and modest sample size. Especially, the timing of WAIS assessment was not fixed and varied among patients because testing was performed when patients were considered able to complete the WAIS reliably. Furthermore, potential confounding factors such as depressive symptoms were not assessed. To overcome these limitations, future studies should include longitudinal outcomes, neurophysiological measures, and multicenter validations to enhance the generalizability.

## Conclusion

In the present study, neuropsychological assessment using the WAIS revealed a selective reduction in PSI in patients with subarachnoid hemorrhage due to rupture of the anterior communicating artery aneurysm, whereas other cognitive domains remained relatively preserved. These findings indicate that administering the WAIS enables the sensitive detection of processing speed impairment and may facilitate the timely implementation of targeted rehabilitation strategies to optimize functional recovery.

Summary of the coefficient values of the multivariable linear regression model with PSI as the dependent variable. Explanatory variables included age, WFNS grade, aneurysm location, days from onset to assessment, WAIS version. Each point represents the unstandardized regression coefficient, with horizontal bars indicating the 95% confidence interval. The vertical dashed line marks the zero-effect reference; variables whose confidence intervals cross this line do not exhibit statistically significant contributions. P-values for each predictor are presented on the right.

## Supplementary Information

Supplementary Material 1.

## Data Availability

Additional clinical information related to the presented cases is available from the corresponding author upon reasonable request.

## Abbreviations

ACC: Anterior cingulate cortex
Acom: Anterior communicating artery
ANOVA: Analysis of variance
CG: Callosal Genu
FH–CR: Frontal Horn–Callosal Rostrum\
FSIQ: Full-Scale Intelligence Quotient
HDS-R: Hasegawa Dementia Scale–Revised
MoCA: Montreal Cognitive Assessment
PRI: Perceptual Reasoning Index
PSI: Processing Speed Index
SAH: Subarachnoid hemorrhage
TMT: Trail Making Test
VCI: Verbal Comprehension Index
WAIS: Wechsler Adult Intelligence Scale
WMI: Working Memory Index

## References

[1] Collaborators G, Rautalin GSHRF, Volovici I, Stark V, Johnson BA, Kaprio CO. J, Global, Regional, and National Burden of Nontraumatic Subarachnoid Hemorrhage: The Global Burden of Disease Study 2021. JAMA Neurol. 2025; Available from: https://jamanetwork.com/journals/jamaneurology/fullarticle/2834680. Cited 25 Jun 2025. 10.1001/jamaneurol.2025.1522 PMC12557468 40406922

[2] Koupaei SRA, Ziaee M, Baharvahdat H, Ahmadi Z, Deluee MT, Kakhki BR et al. An Epidemiological Investigation on Patients with Non-traumatic Subarachnoid Hemorrhage from 2010 to 2020. Bull Emerg Trauma . 2024;12(1):35. Available from: https://pmc.ncbi.nlm.nih.gov/articles/PMC11057451/. Cited 25 Jun 2025. 10.30476/BEAT.2024.101708.1495 PMC11057451 38689795

[3] Wang Z, Liu Y, Qie R, Hu Y. Comparative analysis of stroke burden between ages 20–54 and over 55 years: based on the global burden of disease study 2019. BMC Public Health . 2025 Dec 1;25(1):1293. Available from: https://pmc.ncbi.nlm.nih.gov/articles/PMC11972468/. Cited 23 Nov 2025. 10.1186/s12889-025-22460-6 PMC11972468 40188028

[4] Lv B, Lan JX, Si YF, Ren YF, Li MY, Guo FF et al. Epidemiological trends of subarachnoid hemorrhage at global, regional, and national level: a trend analysis study from 1990 to 2021. Military Medical Research 2024 11:1 . 2024;11(1):46-. Available from: https://mmrjournal.biomedcentral.com/articles/10.1186/s40779-024-00551-6. Cited 2025 Nov 23. 10.1186/s40779-024-00551-6 PMC11241879 38992778

[5] Jorna LS, Khosdelazad S, Kłos J, Rakers SE, van der Hoorn A, Potze JH et al. Automated magnetic resonance imaging quantification of cerebral parenchymal and ventricular volume following subarachnoid hemorrhage: associations with cognition. Brain Imaging and Behavior 2024 18:2 . 2024;18(2):421–9. Available from: https://link.springer.com/10.1007/s11682-024-00855-0. Cited 2025 Nov 23. 10.1007/s11682-024-00855-0 PMC10830824 38294581

[6] Haug T, Sorteberg A, Sorteberg W, Lindegaard KF, Lundar T, Finset A. Cognitive outcome after aneurysmal subarachnoid hemorrhage: Time course of recovery and relationship to clinical, radiological, and management parameters. Neurosurgery . 2007;60(4):649–56. Available from: https://journals.lww.com/neurosurgery/fulltext/2007/04000/cognitive_outcome_after_aneurysmal_subarachnoid.10.aspx. Cited 2025 Nov 23. 10.1227/01.NEU.0000255414.70807.A0 17415201

[7] Nussbaum ES, Mikoff N, Paranjape GS. Cognitive deficits among patients surviving aneurysmal subarachnoid hemorrhage. A contemporary systematic review. Br J Neurosurg . 2021;35(4):384–401. Available from: https://www.tandfonline.com/doi/abs/10.1080/02688697.2020.1859462. Cited 2025 Jun 14. 10.1080/02688697.2020.1859462 33345644

[8] Sousa L, Antunes A, Mendes T, Reimão S, Neto LL, Campos J. Long-term Neuropsychiatric and Neuropsychological Sequelae of Endovascularly Treated Aneurysmal Subarachnoid Hemorrhage. Acta Med Port. 2019;32(11):706–13. 31703183 10.20344/amp.10894

[9] García-Pérez P, Rodríguez-Martínez MC, Gallardo-Tur A, Blanco-Reina E, de la Cruz-Cosme C, Lara JP. Early Occupational Therapy Intervention post-stroke (EOTIPS): A randomized controlled trial. PLoS One . 2024;19(8). Available from: https://pubmed.ncbi.nlm.nih.gov/39159190/. Cited 2025 Nov 23. 10.1371/journal.pone.0308800 PMC11332918 39159190

[10] Xuefang L, Guihua W, Fengru M. The effect of early cognitive training and rehabilitation for patients with cognitive dysfunction in stroke. Int J Methods Psychiatr Res . 2021;30(3). Available from: https://pubmed.ncbi.nlm.nih.gov/34132448/. Cited 2025 Nov 23. 10.1002/mpr.1882 PMC8412226 34132448

[11] Kreiter KT, Copeland D, Bernardini GL, Bates JE, Peery S, Claassen J et al. Predictors of cognitive dysfunction after subarachnoid hemorrhage. Stroke . 2002;33(1):200–8. Available from: https://www.ahajournals.org/doi/pdf/10.1161/hs0102.101080?download=true. Cited 15 Jun 2025. 10.1161/hs0102.101080 11779911

[12] Cahill WJ, Calvert JH, Zhang JH. Mechanisms of early brain injury after subarachnoid hemorrhage. Journal of Cerebral Blood Flow and Metabolism . 2006 Nov 15;26(11):1341–53. Available from: https://scholar.google.com/scholar_url?url=https://journals.sagepub.com/doi/pdf/10.1038/sj.jcbfm.9600283&hl=ja&sa=T&oi=ucasa&ct=ufr&ei=mr9NaNLFEL2W6rQP-8qayAk&scisig=AAZF9b9RrYwvgAQqvE6uXGW4dm_s. Cited 15 Jun 2025. 10.1038/sj.jcbfm.9600283 16482081

[13] J V, P H, J O, A S, O H. Social outcome related to cognitive performance and computed tomographic findings after surgery for a ruptured intracranial aneurysm. Neurosurgery . 1990 Apr;26(4):579. Available from: https://pubmed.ncbi.nlm.nih.gov/2330078/. Cited 15 Jun 2025. 10.1097/00006123-199004000-00004 2330078

[14] Desantis A, Laiacona M, Barbarotto R, Basso A, Villani R, Spagnoli D et al. Neuropsychological outcome of patients operated upon for an intracranial aneurysm: Analysis of general prognostic factors and of the effects of the location of the aneurysm. J Neurol Neurosurg Psychiatry . 1989;52(10):1135–40. Available from: https://pubmed.ncbi.nlm.nih.gov/2795039/. Cited 9 Aug 2025. 10.1136/jnnp.52.10.1135 PMC1031697 2795039

[15] Haug T, Sorteberg A, Sorteberg W, Lindegaard KF, Lundar T, Finset A. Cognitive functioning and health related quality of life after rupture of an aneurysm on the anterior communicating artery versus middle cerebral artery. Br J Neurosurg . 2009;23(5):507–15. Available from: https://pubmed.ncbi.nlm.nih.gov/19718555/. Cited 9 Aug 2025. 10.1080/02688690902785701 19718555

[16] Tidswell P, Dias PS, Sagar HJ, Mayes AR, Battersby RDE. Cognitive outcome after aneurysm rupture: Relationship to aneurysm site and perioperative complications. Neurology . 1995;45(5):876–82. Available from: https://pubmed.ncbi.nlm.nih.gov/7746400/. Cited 9 Aug 2025. 10.1212/wnl.45.5.876 7746400

[17] Schellekens MMI, Boot EM, Verhoeven JI, Ekker MS, Verburgt E, Immens MHM et al. Cognitive performance is associated with return to work after ischemic stroke in young adults: The ODYSSEY study. Eur Stroke J . 2025 Sep 1;10(3):784–95. Available from: https://pubmed.ncbi.nlm.nih.gov/40071576/. Cited 23 Nov 2025. 10.1177/23969873251324400 PMC11907581 40071576

[18] Samuelsson H, Viken J, Redfors P, Holmegaard L, Blomstrand C, Jern C et al. Cognitive function is an important determinant of employment amongst young ischaemic stroke survivors with good physical recovery. Eur J Neurol . 2021 Nov 1;28(11):3692–701. Available from: 10.1111/ene.15014. Cited 23 Nov 2025. 10.1111/ene.15014 34242459

[19] Sánchez-Cubillo I, Periáñez JA, Adrover-Roig D, Rodríguez-Sánchez JM, Ríos-Lago M, Tirapu J et al. Construct validity of the Trail Making Test: role of task-switching, working memory, inhibition/interference control, and visuomotor abilities. J Int Neuropsychol Soc . 2009;15(3):438–50. Available from: https://pubmed.ncbi.nlm.nih.gov/19402930/. Cited 29 Nov 2025. 10.1017/S1355617709090626 19402930

[20] Salthouse TA. What cognitive abilities are involved in trail-making performance? Intelligence . 2011 Jul;39(4):222–32. Available from: https://pubmed.ncbi.nlm.nih.gov/21789028/. Cited 29 Nov 2025. 10.1016/j.intell.2011.03.001 PMC3141679 21789028

[21] Wechsler D. WAIS-III: administration and scoring manual. 3rd ed. San Antonio (TX): Psychological Corporation; 1997.

[22] Wechsler D. WAIS-IV: administration and scoring manual. 4th ed. San Antonio (TX): Pearson; 2008.

[23] Jones AD, Cho RY, Nystrom LE, Cohen JD, Braver TS. A computational model of anterior cingulate function in speeded response tasks: Effects of frequency, sequence, and conflict. Cogn Affect Behav Neurosci. 2002;2(4):300–17. 12641175 10.3758/cabn.2.4.300

[24] Mulert C, Gallinat J, Dorn H, Herrmann WM, Winterer G. The relationship between reaction time, error rate and anterior cingulate cortex activity. International Journal of Psychophysiology . 2003 Feb 1;47(2):175–83. Available from: https://pubmed.ncbi.nlm.nih.gov/12568947/. Cited 30 Nov 2025. 10.1016/s0167-8760(02)00125-3 12568947

[25] Imai Y, Hasegawa K. The Revised Hasegawa’s Dementia Scale (HDS-R): evaluation of its usefulness as a screening test for dementia. J Hong Kong Coll Psychiatr. 1994;4(2):20–4.

[26] Naito E, Kinomura S, Geyer S, Kawashima R, Roland PE, Zilles K. Fast Reaction to Different Sensory Modalities Activates Common Fields in the Motor Areas, but the Anterior Cingulate Cortex is Involved in the Speed of Reaction. https://doi.org/101152/jn20008331701 . 2000;83(3):1701–9. Available from: /doi/10.1152/jn.2000.83.3.1701. Cited 14 Sep 2025. 10.1152/jn.2000.83.3.1701 10712490

[27] He C, Rong S, Zhang P, Li R, Li X, Li Y et al. Metabolite changes in prefrontal lobes and the anterior cingulate cortex correlate with processing speed and executive function in Parkinson disease patients. Quant Imaging Med Surg . 2022 Aug 1;12(8):4226–38. Available from: https://pubmed.ncbi.nlm.nih.gov/35919059/. Cited 10 Aug 2025. 10.21037/qims-21-1126 PMC9338382 35919059

[28] Vallesi A, Mcintosh AR, Crescentini C, Stuss DT. fMRI investigation of speed–accuracy strategy switching. Hum Brain Mapp . 2011 Jul;33(7):1677. Available from: https://pmc.ncbi.nlm.nih.gov/articles/PMC6870457/. Cited 10 Aug 2025. 10.1002/hbm.21312 PMC6870457 21618664

[29] Eckert MA, Keren NI, Roberts DR, Calhoun VD, Harris KC. Age-related changes in processing speed: Unique contributions of cerebellar and prefrontal cortex. Front Hum Neurosci . 2010 Mar 8;4:1178. Available from: https://www.frontiersin.org. Cited 10 Aug 2025. 10.3389/neuro.09.010.2010 PMC2839847 20300463

[30] Bowie CR, Harvey PD. Administration and interpretation of the Trail Making Test. Nat Protoc . 2006 Dec;1(5):2277–81. Available from: https://pubmed.ncbi.nlm.nih.gov/17406468/. Cited 29 Nov 2025. 10.1038/nprot.2006.390 17406468

[31] O’Bryant SE, Humphreys JD, Smith GE, Ivnik RJ, Graff-Radford NR, Petersen RC et al. Detecting dementia with the mini-mental state examination in highly educated individuals. Arch Neurol . 2008 Jul;65(7):963–7. Available from: https://pubmed.ncbi.nlm.nih.gov/18625866/. Cited 29 Nov 2025. 10.1001/archneur.65.7.963 PMC2587038 18625866

[32] Nasreddine ZS, Phillips NA, Bédirian V, Charbonneau S, Whitehead V, Collin I et al. The Montreal Cognitive Assessment, MoCA: a brief screening tool for mild cognitive impairment. J Am Geriatr Soc . 2005;53(4):695–9. Available from: https://pubmed.ncbi.nlm.nih.gov/15817019/. Cited 29 Nov 2025. 10.1111/j.1532-5415.2005.53221.x 15817019

