# Supplementary material for: Processing speed impairment after anterior communicating artery aneurysm rupture assessed using the wechsler adult intelligence scale: an observational study
Source: BMC Neurol. 2026 Apr 15;26:342. doi: 10.1186/s12883-026-04902-9 (PMC13191877; doi:10.1186/s12883-026-04902-9)
Supplement: Supplementary file 1 — Supplementary Material 1. [file 12883_2026_4902_MOESM1_ESM.pdf]

**Supplementary Materials for**
'Processing Speed Impairment After Anterior Communicating Artery Aneurysm Rupture Assessed Using the Wechsler Adult Intelligence Scale: An Observational Study'

Takuya Okui[1, †], Natsuko Otani[2, †], Takamitsu Iwata[1]*, Koichi Beppu[1], Ryo Horii[1], Ryuichiro Kajikawa[1], Takashi Tsuzuki[1]

Author affiliations:
[1] Department of Neurosurgery, Sakai City Medical Center, Sakai, Osaka, Japan
[2] Department of Rehabilitation Technology, Sakai City Medical Center, Sakai, Osaka, Japan
[†]These authors contributed equally to this work.

**This PDF file includes the following**:

Supplementary Figures 1 to 4

Supplementary Tables 1

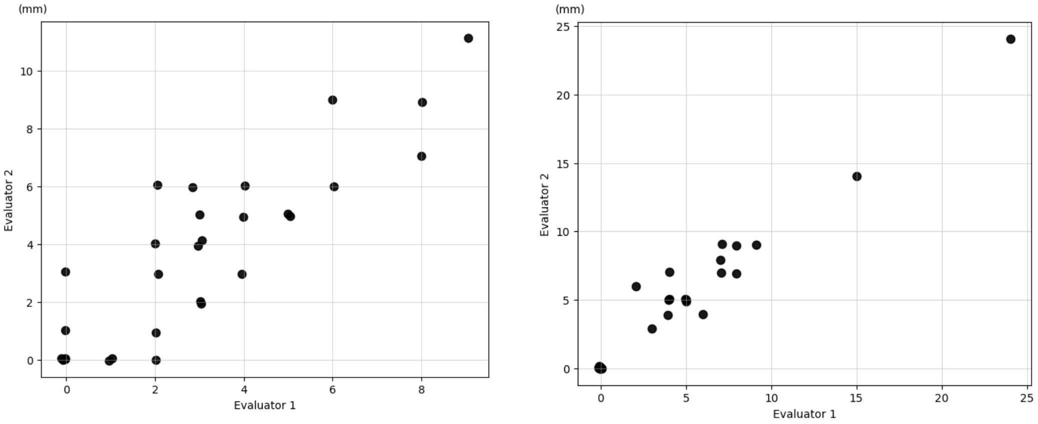


Supplementary figure1 **Inter-rater reliability of interhemispheric hematoma thickness measurements.**

Hematoma thickness was independently measured at the FH–CR and CG planes by two raters in a blinded manner (blinded to clinical information, aneurysm location, and neuropsychological results). Inter-rater agreement was excellent, assessed using a two-way random-effects intraclass correlation coefficient for absolute agreement, single measurement [ICC(2,1)]: ICC(2,1) = 0.839 (95% CI 0.680–0.918) at the FH–CR plane and ICC(2,1) = 0.976 (95% CI 0.896–0.995) at the CG plane.

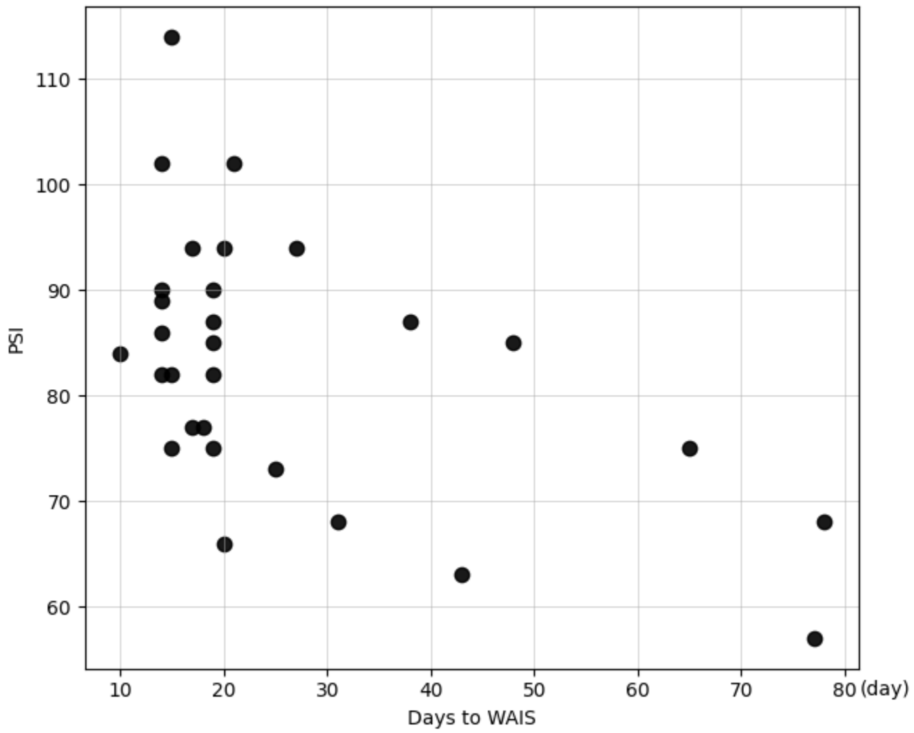


Supplementary figure2 **Relationship between processing speed and time from onset to WAIS assessment.**

Scatter plot showing the association between the Processing Speed Index (PSI) and days from SAH onset to WAIS assessment (x-axis: days-to-test; y-axis: PSI). PSI demonstrated a moderate negative correlation with time-to-assessment ($r = -0.531$, $p = 0.0030$; Pearson's correlation test).

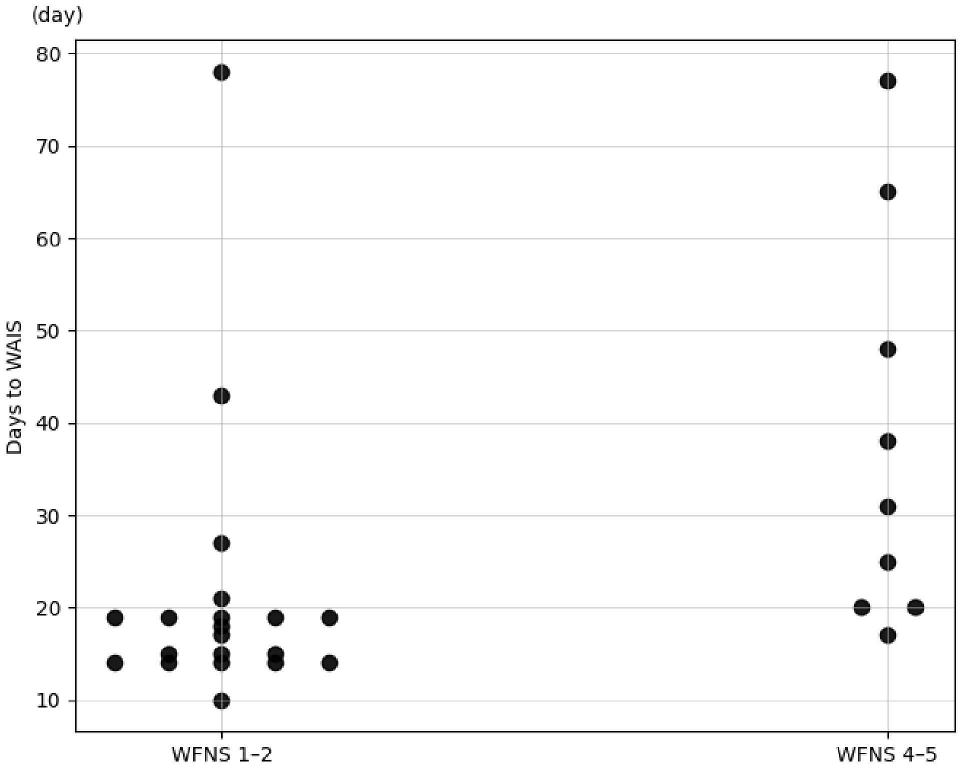


Supplementary figure3 **Comparison of time-to-assessment between mild and severe SAH groups.**

Days from SAH onset to WAIS assessment were compared between the mild (WFNS grades 1–2) and severe (WFNS grades 4–5) groups. The severe group was assessed significantly later than the mild group (mild: n = 20, median [IQR] 17.5 [14.0–19.0] days; severe: n = 9, median [IQR] 31.0 [20.0–48.0] days; Mann–Whitney U test, p = 0.00383).

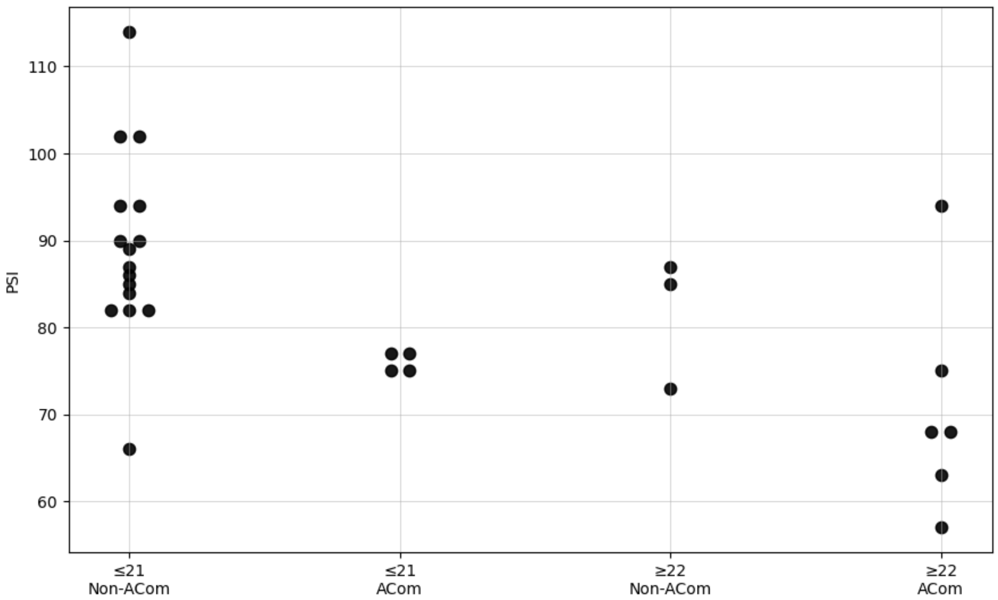


Supplementary figure 4 **Time-stratified sensitivity analysis of PSI by aneurysm location.**

Processing Speed Index (PSI) was compared between the Acom and non-Acom groups after stratifying patients by the time from SAH onset to WAIS assessment (≤21 days vs ≥22 days). In the ≤21-day subgroup (n = 20), PSI was significantly lower in the Acom group (Acom: n = 4, mean 76.00 [SD 1.15] vs non-Acom: n = 16, mean 89.31 [SD 10.78]; Welch's t-test, p = 0.00018), consistent with the primary analysis. In the ≥22-day subgroup (n = 9), PSI remained numerically lower in the Acom group (Acom: n = 6, mean 70.83 [SD 12.83] vs non-Acom: n = 3, mean 81.67 [SD 7.57]), but the difference was not statistically significant (Welch's t-test, p = 0.1596), likely due to limited sample size and reduced statistical power.

| | β | SE | t | p | 95%CI low | 95%CI high |
|---|---|---|---|---|---|---|
| age | -0.074 | 0.198 | -0.375 | 0.712 | -0.493 | 0.344 |
| WFNS | -2.749 | 1.875 | -1.467 | 0.1607 | -6.704 | 1.206 |
| Days to WAIS | -0.065 | 0.164 | -0.396 | 0.6973 | -0.412 | 0.282 |
| Acom | -11.369 | 5.367 | -2.119 | 0.0492 | -22.692 | -0.047 |

Supplementary Table1 **WAIS-IV-only multivariable regression analysis predicting PSI.**

A sensitivity analysis was performed restricting the cohort to patients assessed with WAIS-IV (n = 22). Multivariable linear regression was conducted using the prespecified model: PSI ~ age + WFNS + days-to-WAIS + Acom. Acom location remained the only significant negative predictor of PSI ($\beta = -11.37$, 95% CI −22.69 to −0.05, $p = 0.049$), whereas age, WFNS grade, and days-to-WAIS were not significant. Model fit was $R^2 = 0.426$ (adjusted $R^2 = 0.291$).
